# A theory-based educational intervention targeting nurses' attitudes and knowledge concerning cancer-related pain management: A study protocol of a quasi-experimental design

**DOI:** 10.1186/1472-6963-11-233

**Published:** 2011-09-23

**Authors:** Gunilla Borglin, Markus Gustafsson, Hans Krona

**Affiliations:** 1 School of Health Science, Blekinge Institute of Technology, SE-379 71 Blekinge, Sweden

## Abstract

**Background:**

Pain is one of the most frequent problems among patients diagnosed with cancer. Despite the availability of effective pharmacological treatments, this group of patients often receives less than optimal treatment. Research into nurses' pain management highlights certain factors, such as lack of knowledge and attitudes and inadequate procedures for systematic pain assessment, as common barriers to effective pain management. However, educational interventions targeting nurses' pain management have shown promise. As cancer-related pain is also known to have a negative effect on vital aspects of the patient's life, as well as being commonly associated with problems such as sleep, fatigue, depression and anxiety, further development of knowledge within this area is warranted.

**Methods/design:**

A quasi-experimental study design will be used to investigate whether the implementation of guidelines for systematic daily pain assessments following a theory-based educational intervention will result in an improvement in knowledge and attitude among nurses. A further aim is to investigate whether the intervention that targets nurses' behaviour will improve hospital patients' perception of pain. Data regarding nurses' knowledge and attitudes to pain (primary outcome), patient perception regarding pain (secondary outcome), together with socio-demographic variables, will be collected at baseline and at four weeks and 12 weeks following the intervention.

**Discussion:**

Nursing care is nowadays acknowledged as an increasingly complicated activity and "nursing complexity is such that it can be seen as the quintessential complex intervention." To be able to change and improve clinical practice thus requires multiple points of attack appropriate to meet complex challenges. Consequently, we expect the theory-based intervention used in our quasi-experimental study to improve care as well as quality of life for this group of patients and we also envisage that evidence-based guidelines targeting this patient group's pain will be implemented more widely.

**Trial Registration Number:**

ClinicalTrials.gov NCT01313234

## Background

Pain is one of the most common problems among patients suffering from cancer [1]. Despite the availability of effective pharmacological treatments and the fact that 70 - 90% of the patients can gain pain relief with the correct pain management, this group often receives less than optimal treatment [2-7]. This is demonstrated, for example, in one of the most extensive studies performed in Europe (EPIC), which found that 43% of the Swedish patients experienced cancer-related pain, 18% of whom did not receive any pain-relieving medication, despite grading their pain as moderate to severe on the Visual Analogue Scale (VAS ≥ 5) [8]. In a Meta-analysis (52 studies included) by van den Beuken and colleagues [1] investigating the prevalence of cancer-related pain, 64% of those with advanced cancer, 59% of those receiving cancer treatment and 33% of those after treatment experienced some degree of pain. Additionally, more than one-third graded their pain as moderate to severe [1]. These studies consequently imply that there is still some work that needs to be done for nurses within the area of cancer-related pain management. Furthermore, we know that cancer-related pain can have a negative impact on several vital aspects of the patient's life and cancer-related pain is commonly associated with other problems, such a sleep, fatigue, depression and anxiety [9,10]. Subsequently, cancer-related pain is an important problem to target in health service research. In-depth interviews [8] have shown that patients sometimes experience their cancer-related pain as being so difficult that they say they "would rather die" and that their nurse or doctor never or rarely asks them about their pain. All of the above are serious issues, which are prone to result in reduced quality of life for this group of patients. Interventions targeting the implementation of acceptable pain management in clinical practice are thus critical in the nursing care of these patients.

Studies investigating barriers to adequate pain management among registered nurses (RN) have identified lack of knowledge of cancer-related pain and pain treatment [11,12] as a serious obstacle to acceptable pain relief. The RNs' attitude to pain and pain treatment, i.e. their own subjective judgement about the patient's pain, such as reliance on non-verbal behaviour as a pain indicator, has also been shown to impede appropriate pain management [13]. Research identifies other plausible explanations for inadequate treatment of cancer-related pain, such as insufficient routine procedures for systematic measurement and assessment of pain [14,15]. Consequently, the introduction of pain assessment instruments has been shown to reduce the impact of pain on patients' daily lives as well as improving how they manage their pain [16]. The Swedish Society of Nursing has developed national guidelines for pain assessment of cancer-related pain, recommending that pain should be assessed routinely and systematically. Despite the existence of such national guidelines it is not yet standard practice on general hospital wards in Sweden to screen for pain or to systematically assess pain among patients on a daily basis.

Attitudes to and knowledge of pain and its treatment can be improved by means of educational interventions [17,18], and this will also contribute to eliminating the RNs' barriers to pain management [19]. However, when designing educational interventions research indicates that it is important to bear in mind how human nature works in relation to behavioural change [20,21], i.e. change of practice, and that possible positive improvement in behavioural change may not be sustained [17]. It is also well known that education *per se *is not an effective and viable strategy for changing behaviour. Instead, interactive learning in small groups and learning from personal experience have proved to be winning blueprints for change [22]. To successfully bring about behavioural change, the educational strategies also need to enhance feelings of ownership and initiate involvement from those participating.

Our review of the literature has highlighted the importance of a change in nursing practice, but also in attitudes and knowledge among RNs about cancer-related pain management in general. To improve the quality of care in this group of patients further investigations into this complex of problems are thus warranted.

## Methods/Design

### Study questions

1) Will an intervention consisting of the implementation of guidelines on daily systematic pain assessment following a theory-based education, targeting cancer-related pain and pain treatment, lead to a significantly positive improvement in RNs' knowledge and attitudes regarding their pain management?

2). Will the interventions targeting RNs influence hospital patients' perception of their cancer-related pain?

### Study design

**Figure 1:**
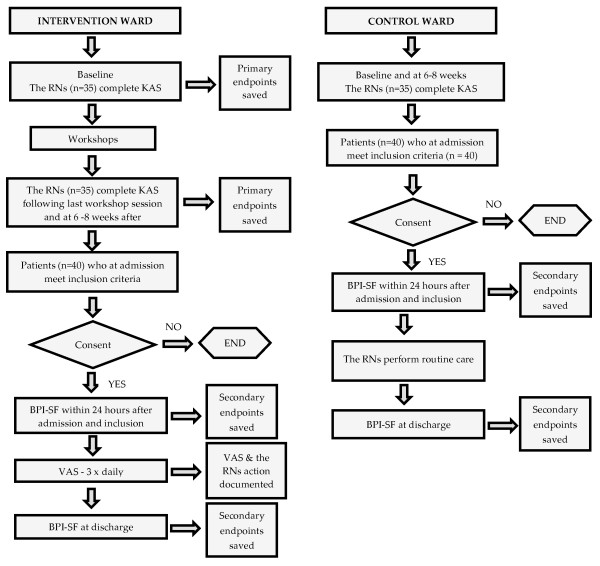
**Overview of study design**.

This study will be carried out with a quasi-experimental design with non-equivalent control groups (Figure 1). Our rationale for a quasi-experimental design was that this type of design is known to be useful for testing causal hypotheses in field settings. It is acknowledged as a practical and feasible design while providing a systematic framework for answering questions relevant to 'real' clinical practice [23]. The design is also considered to be less intrusive on conditions in natural settings. It is important to acknowledge that due to the lack of randomisation, the intervention and control group may be systematically different. However, as it was not possible within the scope of this study to achieve a true experimental design a quasi-experimental design with non-equivalent control groups where the intervention can be separated from effects due to extraneous variables is preferred [23].

### Setting, sample and sample size considerations

Participants (i.e. patients and RNs) will be recruited from two surgical wards specialising in patients with a cancer diagnosis at a hospital in South East Sweden. The hospital has 370 beds and provides services for approximately 150,000 people in the region. The intervention and control ward (26 beds on each ward) has 70 RNs and each year the wards admit around 820 patients who have been diagnosed with cancer.

Following negotiations on all organisational levels, both wards have agreed to participate in the study. The reason for this optimal numbers of participating RNs (n = 70) is that this study is seen within the organisation as providing support to the staff's annual education slots but also as part of supporting quality assurance of its care provision. Assignment to either the intervention ward or control ward is done by the research team.

Sample size calculations for the primary outcome measured by the Knowledge and Attitudes Survey Regarding Pain (KAS) are based on an expected 4.8 change in scores [24] among the RNs on the intervention ward and no changes among the RNs on the control ward. A significance level ᾳ of 0.05 (two-sided) and a power of 80% would require 33 RNs per group. Sample size calculations for the secondary outcome measure, the BPI-SF are based on the expectancy of a 1, 8 point decrease in total score regarding item current pain intensity among the patients on the intervention ward compared to the patients on the control ward [25]. Based on a significance level ᾳ of 0.05 (two sided) and a power of 95% this would require 30 patients per group.

### Inclusion criteria

We will include all RNs on the control ward and intervention ward in accordance with what is stated above. We will include patients with a cancer diagnosis who are aged 18 years and older and who are cognitively intact and able to communicate verbally. We will establish the presence of cancer-related pain for the last 24 hours using the VAS [26] at time of admission or no later than within the first 24 hours following admission. Patients with a score of > 1 on the VAS will be invited to participate.

### Exclusion criteria

RNs coming to work on the intervention ward or control ward as temporary staff will be excluded from participating in the study. We will exclude patients admitted onto the ward because of trauma or planned and/or acute surgery.

#### Intervention programme

Our intervention will focus two key components: (1) a theory-based education for ward RNs caring for patients with a cancer diagnosis and (2) the introduction and implementation of guidelines for daily, systematic pain assessments for those patients on the wards suffering from cancer-related pain.

##### (1) Education - workshops

The educational intervention for nurses will be arranged in the form of workshops. In this study we have defined the concept of workshop as an interactive activity aimed at promoting learning about a pre-set subject or topic. Workshops are said to support the participants in the process of jointly creating insight, knowledge and understanding. It is a democratic and social process [19] created by the interactions that occur between the facilitator and those participating. This results in all competencies present acting as resources in the creation of learning.

**Figure 2:**
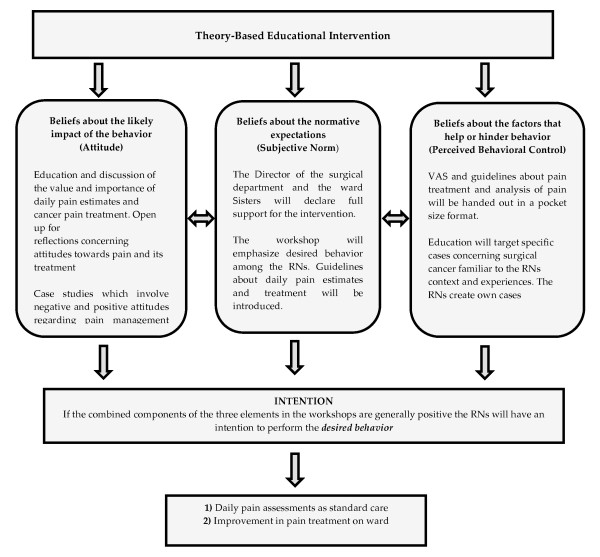
**Overview of theory based intervention - workshops**.

The content of the workshops will be based on the Scottish Intercollegiate Guidelines Network for the treatment of cancer pain in adults [27], and on the literature search performed for this study. As we are aiming for sustained behavioural change among the RNs the curriculum for the workshops will be developed from the Theory of Planned Behaviour [19]. Consequently, the curriculum will include the three distinct elements - beliefs about the likely impact of the behaviour, beliefs about the normative expectations and beliefs about the factors that help or hinder behaviour - which Ajzen [19] suggests controls human behaviour. Importantly, TPB describes intention as the combined result of the three elements i.e. the individual's attitudes, subjective norms, and perceived behavioural control. If these are generally positive, the individual will have the intention of performing the behaviour and if the behaviour is beyond the individual's control the behaviour does not occur. Perceived behavioural control thus has a direct impact on behaviour. As a result, the training in the workshops will focus on these components to achieve greater intention (Figure 2).

The educational intervention consists of a set of workshops (180 minutes each) and each workshop will include 15 RNs from the intervention ward. The first workshop will comprise sessions on pain anatomy, physiology and pain analysis through case study discussions. The second workshop will comprise subjects such as different treatments for cancer-related pain and case study discussions (Figure 2). The workshops will be conducted partly in working groups but also as free discussions in the group as a whole. According to Ajzen [28], persuasive communication theory can also be used to change a form of behaviour. To maintain possible positive behaviour following the intervention, reminder tags will be set up on a regular basis to inform nurses about the most recent guidelines for pain management.

##### (2) The implementation of guidelines concerning daily systematic pain assessments

Guidelines for pain management and systematic pain assessment will be implemented on the intervention ward immediately following the educational intervention. The daily pain assessment will be integrated as a required standard parameter together with the ordinary routine vital parameters such as temperature, pulse and blood pressure. Routine vital parameters on the wards are performed by RNs representing three different work shifts and pain will thus be assessed by the VAS at three points in time i.e. 4 am - 6 am, 1 pm - 3 pm and 7 pm - 9 pm. Pain intensity, nursing action and nursing follow up will be documented in the patient-specific study protocol at the bedside.

VAS is an instrument consisting of a 10 cm horizontal line, which measures pain intensity from "no pain to "worst pain imaginable". Patients participating in the study will be asked to assess current pain intensity by marking the line between the two extremes [26]. The average test-retest coefficient across four studies involving adults with cancer-related pain was r = 0.80 [29]. VAS can be used as a regular, short-term assessment instrument for pain intensity as well as an evaluation of ongoing pain treatment [30].

#### Control intervention - usual care

Patients with a cancer diagnosis admitted to the control ward will receive the usual care from their RNs, i.e. care for their patients according to normal practice.

#### Outcome parameters

##### Primary outcome measure

Our primary outcome will be RNs' attitude and knowledge as measured using a modified version of the KAS [31] at baseline, four weeks and twelve weeks after the intervention for the RNs on the intervention ward (Figure 1). As there is a risk that the RNs on the control ward may be inadvertently exposed to the interventions, i.e. assessing their patients' pain intensity and pain impact on daily life, the RNs on the control ward will also be assessed at baseline and at twelve weeks to check for exposure biases.

The original KAS consists of 40 items where items 1-22 are false-true statements, items 23-36 are multiple-choice questions and items 37-40 consist of two case studies [31]. KAS has shown test-retest reliability (r > 0.80) [31] and internal consistency [32] range between 0.70 - 0.73 [31,33]. KAS takes around 10-15 minutes to complete and the number of correct answers is divided by the number of items (38) to produce the percentage of the total score [31]. We have removed two items as they were considered to be context-specific for the instrument's country of origin. The instrument has not been used in Sweden previously, hence a back translation by an authorised translator from English to Swedish was made.

##### Secondary outcome measure

We will measure pain intensity and pain impact on daily life among the patients using the Brief Pain Inventory - Short Form [34]. The patient assessments by BPI-SF on admission (or no later than 24 hrs after admission) to the wards (intervention ward and control ward) and on discharge from the wards will follow directly after the educational intervention for the RNs on the intervention ward and control ward (Figure 1). The BPI-SF will be undertaken by the RNs admitting, discharging and/or responsible for the patient. Our rationale for including assessments of the patients on the control ward is our wish to rule out competing explanations for results obtained [23], i.e. a positive change in the patients' pain perception on the intervention ward. Hence, both groups will be compared in terms of secondary outcome, demography and diagnosis.

BPI-SF is a pain assessment instrument developed for cancer patients [34]. It is widely used and demonstrates sound psychometric properties [2,4,35-42]. The Swedish version has been linguistically validated but has not yet been psychometrically validated [43]. The instrument consists of nine items, as well as a figure depicting a human body where the patient marks the location and type of pain. BPI-SF includes items relating to the current degree of pain, pain during the last 24 hours and pain on average, as well as items related to the effect of pain treatment, walking ability, mood, work, relationships and whether sleep is affected by pain [34].

### Statistical analysis

Comparability between intervention and control groups will be assessed at baseline and at end of study to check for differences between participants, i.e. RNs and patients. Outcomes (KAS and BPI-SF) together with variables such as demographics, diagnosis, age, RN's length of experience and education, both at baseline and at end of study, will be compared between the intervention and control groups using both univariate and multivariate techniques [44]. Data will be analysed in accordance with the intention-to-treat principle. All participants with valid data regardless of whether they remained in the setting at baseline, drop-outs and losses to follow-up will be described.

### Ethical issues

We will conduct the study in compliance with the established ethical guidelines of the Declaration of Helsinki [45]. Under the Swedish Ethical Review Act (2003:460) [46] this study does not need ethical clearance by a Regional Ethical Review Board, although we have nevertheless sought and received ethical guidance and advisory opinions from the Ethical Advisory Board in South-East Sweden (ref. 61-2011). All participants will receive verbal and written information about the study and will be informed of their right to withdraw at any time. To ensure compliance with the Data Protection Act [47], data will be stored securely and anonymised and only the research team will have access to the data. No published material will contain patient-identifiable information.

#### Obtaining informed consent from participants

Patients who meet the inclusion criteria on both participating surgical wards will be invited to take part in the study by the admitting RN or by the RN in charge of the patient. Both written and verbal information about the study and its protocol will be provided. All RNs on both wards will be informed verbally and in writing about the study before participating.

#### Risk and anticipated benefits for participants

This project will train RNs in the field of cancer-related pain and establish routines for pain assessment, which is good. Allowing the nurses to update their knowledge in cancer-related pain management may prove beneficial to other groups of patients experiencing pain. The findings of this study will be used to update present guidelines concerning cancer-related pain management. At present, no known risk can be envisaged concerning the patients' physical and/or mental health.

#### Forecast completion date

This study is due to be completed end of December 2011.

## Discussion

Nursing care is nowadays acknowledged as an increasingly complicated activity and "nursing complexity is such that it can be seen as the quintessential 'complex intervention" [48]. To be able to change and improve clinical practice thus demands multiple points of attack to meet complex challenges. Consequently, this paper presents a quasi-experimental study design that aims to i) endorse and assist behavioural change among nurses with regard to cancer-related pain and pain management and ii) change routine practice by implementing daily pain assessments. We expect the theory-based intervention to improve patient care as well as the quality of life for this group of patients but we also envisage that evidence-based guidelines targeting this patient group and their pain will be implemented more widely.

## List of abbreviations used

EPIC: European Pain in Cancer; KAS: Knowledge and Attitudes Survey Regarding Pain; RCT: Randomised Controlled Trial; RN: Registered Nurse; TPB: Theory of Planned Behaviour; VAS: Visual Analogue Scale.

## Competing interests

The authors declare that they have no competing interests.

## Authors' contributions

MG, GB and HK conceived and designed the study and obtained funding. GB and MG drafted the manuscript and HK contributed to editing the final manuscript. All authors read and approved the final manuscript.

## Pre-publication history

The pre-publication history for this paper can be accessed here:

http://www.biomedcentral.com/1472-6963/11/233/prepub
